# Improving service provision through change management

**DOI:** 10.4102/safp.v65i1.5602

**Published:** 2023-01-12

**Authors:** Mergan Naidoo

**Affiliations:** 1 Discipline of Family Medicine, School of Nursing and Public Health, University of KwaZulu-Natal, Durban, South Africa

**Keywords:** change management, theory of change, leadership, management, clinical service

## Abstract

The change agent role of the family physician is often a daunting task with very little guidance on how to approach change leadership in the current fellowship curriculum. This continuing medical education resource will utilise the theory of change and provide some guidance to taking on this task in the workplace. The approach can be used in healthcare, the community, academia, and research projects. The resource will outline a systematic approach to developing a logic-based strategy for outcomes. The process will be unpacked, the evaluation method outlined, and strategies for ensuring the sustainability of the changing culture will be discussed.

## Introduction

Winston Churchill said, ‘To improve is to change; to be perfect is to change often’. That phrase is very relevant for many of us who fulfil the role of clinician, community advocate, educator, researcher and manager. There is a constant need to manage change in the setting of service improvement, community empowerment, managing a research project, curriculum enrichment exercises and improving operational efficiency. This continuing medical education (CME) resource will outline a generic approach that may be useful for the primary care physician who performs many roles in their daily life. There is no formula for implementing change management because work environments are complex and need multiple interactions with people. Hence, it is essential that, as a leader, one is receptive to adapting one’s approach to suit the context. Change is often because of external influences such as oversight visits and new technologies or changes in the institutional climate such as the coronavirus disease 2019 (COVID-19) pandemic and its impact on healthcare delivery, teaching and research.^1^

Change management can involve redesigning the strategy (vision and mission), the structural elements (organogram), the processes (workflow), the personnel (deployment) and the technology. The theoretical unpinning will utilise the theory of change (ToC).^2^ Weiss developed the idea as an evaluation model in the 1990s.^3^ Since then, the model has been utilised extensively in healthcare. Although the approach has been refined over the last two decades, the key is identifying why the issue being addressed is important. The critical quality control criteria include plausibility, feasibility and testability, with plausibility being fundamental. These measures relate to the logic of the outcomes pathways, realistically achieving the long-term outcomes and impact and using measurable indicators, respectively.^4^ The logic model therefore serves as a useful conceptual model when implementing change.

## Unpacking the process (methods)

**FIGURE 1:**
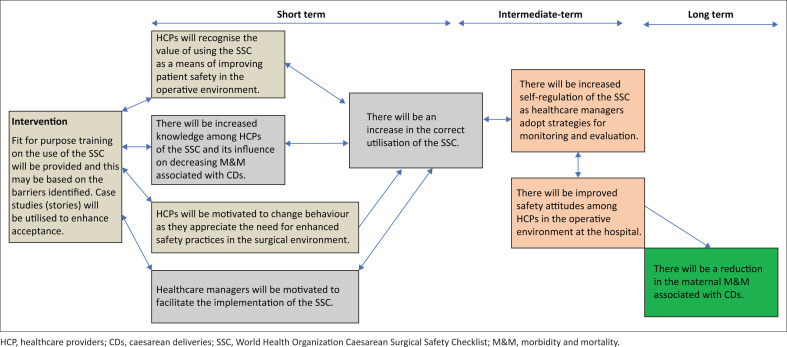
An example of an outcomes chain.

The first step is to ensure that the project has a sound rationale. One should identify why the problem is essential, describe the context, articulate how addressing the issue improves service provision and explain what is different or new about the intervention. The next step is to describe the intervention and relate this to the needs assessment. Based on relevant literature, identify what is known about the problem in similar contexts, what is not yet known, and the results of similar interventions. Determine the project’s overall purpose (vision), define the short, intermediate, and long-term outcomes and outline the project’s impact in 5–10 years. The team will need to identify what short-, intermediate- and long-term translates into time frames. Importantly plan who and what will change. Create a diagram showing the relationship between one’s outcomes (outcomes chain). The outcomes can be based on the literature review and other information obtained through the baseline assessment in defining the issue.^5^ We created an outcome chain when we found the suboptimal implementation of the modified World Health Organization Caesarean Surgical Safety Checklist (SSC) in various hospitals in KwaZulu-Natal.^6^ The long-term impact was that the SSC would reduce maternal morbidity and mortality (M&M). Figure 1 provides an outline of the outcomes chain for this project.

Furthermore, describe how one will go about achieving the outputs and outcomes. The project logic model must specify the resources required (inputs), the activities undertaken, the outcomes (short and intermediate) and the impact (long-term). Short-term outcomes are sometimes called outputs. Inputs include human resources, finances, infrastructure and equipment. Activities define what the project does with the resources to fulfil its vision.^7^ Use specific line items to describe expenses associated with each activity that requires budgetary resources. Determine the project activities and create a Gantt chart.^8^ Each task takes up a row, and completion dates are reflected in increments of time units.^9^ Tasks must be put into a logical order to avoid errors of reason. For example, the printing of the SSC must occur before implementation. Activities are linked to outcomes and should include the people responsible for the task, the time frame and the resources needed.

**FIGURE 2:**
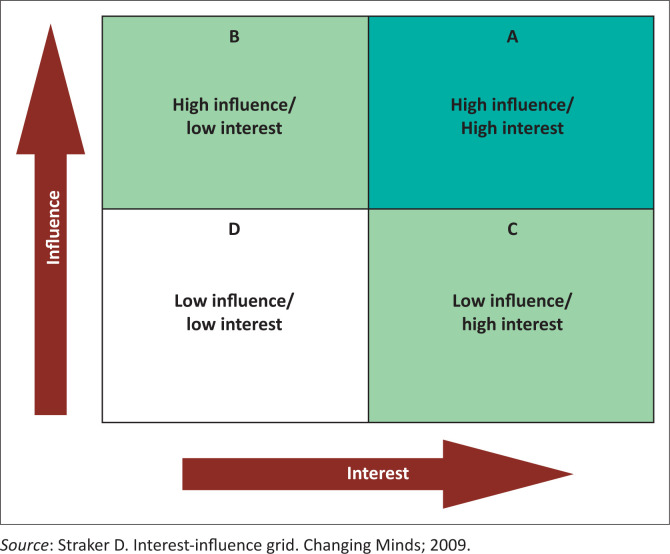
The interest and influence grid.^11^

Define the project team and then outline the project feasibility and the expected deliverables in the first few years. It is always crucial to form a powerful project team and a group of advisors. When creating the project team, it is helpful to use the interest and influence grid (see Figure 2).^10^ Look at each individual in the group and define where they fit in the grid (A-D), their level of commitment to the project, their role (planning team, advisor, consultant, positive energy network) and each stakeholder’s personal motivation (What is in it for me?). Personal motivation may include networking, taking up a challenge, enjoyment, collaboration, co-author on a publication, opportunity to make a difference, visibility, developing new skills, political gain and improving one’s professional portfolio.^5^

During change management, anticipated barriers from stakeholders include denial and resistance. Sometimes the issues arising from such obstacles are insurmountable, causing the project to collapse. The strategies for dealing with such obstacles are as follows: (1) Provide evidence of the need for change and ask for a response based on the information provided, (2) expose stakeholders to learning opportunities that expose them to new practices and ask for feedback, (3) form task teams that explore the best practices, (4) ask members of the team to visit other sites for benchmarking, (5) if there is resistance, create opportunities to express fears and doubts and display empathy, (6) employ active listening and resist the impulse to defend, (7) build a supportive coalition and find individuals who can influence resisters, and (8) explain how the future will change with the intervention.

One must communicate the project’s vision to influential people in the organisation who are often very busy. One strategy is to develop a 2-min elevator speech outlining the purpose, the picture, the plan and what each person will be doing. Write it down, practise it and then seek the 2-min opportunity in the corridor if you cannot get an appointment. Stakeholders are individuals interested in the project or have an influence on the institution and can affect the project positively or negatively. They also have knowledge that can assist with the project or can impact the project. Stakeholders can also be impacted by the project as this may change the way they practise in the organisation. Stakeholders can invest in the project (financial or emotional) or be innovators who can bring new approaches to the tasks.^10^

Key elements of project planning are anticipating possible success factors and challenges. Success factors involve individual, institutional and system strengths that allow the team to achieve their outcomes. Challenges are obstacles that need to be overcome to achieve the results. Using the individual and system strengths, devise ways to overcome the challenges. Identifying strengths and weaknesses in team members’ personalities may provide ideas of personal strengths. Strength-based leadership provides three ingredients for effective leadership: knowing your strengths, building talented teams and meeting the needs of your followers.^12^ Some of the personal strengths of leaders include executing, influencing, building relationships and strategic thinking.

Although many approaches to change management exist, Kotter’s eight-stage model is widely used and cited.^13^ The eight steps are listed here. Critical to all change management initiatives is to get ‘buy-in’ from all the key role players.

- Establishing a sense of urgency involves inspiring people to move and making objectives feasible and relevant.
- Creating a guiding coalition involves getting the right people in place with exemplary emotional commitment and the right mix of skills and levels.
- Developing a change vision requires the team to establish a simple vision and strategy, focusing on emotional and creative aspects necessary to drive service and efficiency.
- Communicating the vision for buy-in involves as many people as possible, sharing the essentials, and appealing and responding to people’s needs. Simplify communications and make technology work for you rather than against you.
- Empowering broad-based action involves removing obstacles and enabling constructive feedback and lots of support from leaders to reward and recognise progress and achievements.
- Generating short-term wins sets accessible, achievable aims in bite-size chunks. Have a manageable number of initiatives. Finish current stages before starting new ones.
- Never letting up involves fostering and encouraging determination and persistence; ongoing change encourages progress reporting and highlights achievements and future milestones.
- Incorporating changes into the culture involves reinforcing the value of successful transition via recruitment, promotion, and new change leaders. Weave change into the organisational culture.

Kotter’s model can be incorporated into contextually driven approaches to improve the success factors.

## Programme evaluation

The programme evaluation must be built into every project a priori. The evaluation may not focus on the overall impact of the project. Still, it may focus on the short-term outcomes and the settling down period to identify issues that are not working well and provide solutions for support and corrective action.^1^ The evaluation plan allows for essential data to be collected appropriately as the project unfolds and to define how data will be collected to evaluate the programme’s efficacy. Data can be gathered using both qualitative and quantitative methods. Some methods may include surveys, interviews and focus group discussions.

Programme evaluation requires answers to the following questions:

- Did it work? Formulate questions about whether the intervention achieved the desired results. Select several outcomes from the outcomes chain and write these as questions. Define specific measurements that will answer these questions. An example would be a greater uptake of using the SSC correctly that translates into better quality of surgical care during a CD. See outcome 3,4, 5 shown in Table 1.
- Why did it work? Formulate questions that explore the ToC. Do this by defining questions that seek to understand how facilitating factors allowed one to achieve the outcomes. Finally, determine specific measurements that will indicate answers to your questions. This may relate to outcome 1 and 5 shown in Table 1.
- What was done? These questions seek to describe the process of the intervention. Do this by selecting a few activities and formulating questions related to these activities. Finally, determine specific measurements that will indicate answers to your questions. This may relate to outcome 6 shown in Table 1.

**TABLE 1:** Data matrix for programme evaluation.

Outcomes	Questions	Indicators	Data sources	Data collection methods
1. Healthcare managers are motivated to facilitate the implementation of the SSC.	Are managers motivated to improve the implementation of the SSC?	Managers’ perception of their motivation to implement.	Transcription from the recorded interview with the QA manager.	Interview with QA manager
2. There will be an increase in the correct utilisation of the SSC.	How much did the utilisation increase?	At least 70% of cases use the SSC correctly (90% compliance with all elements of the SSC).	Extraction from the theatre notes.	The SSC will be inspected from each file to ascertain the number of cases in which it was used and the number of cases that were correctly filled in.
3. There will be improved safety attitudes among HCPs in the operative environment at the hospital.	How much did the intervention improve safety attitudes in the operative environment at the hospital?	The safety attitudes will improve by 20%.	HCPs	Safety attitudes questionnaire pre and post-intervention.
4. There will be a reduction in the maternal M&M resulting from CDs.	How much will the maternal M&M decrease after the implementation of the SSC?	The maternal morbidity associated with CDs will be reduced by 20%.	Extraction from the theatre and clinical notes.	Extraction of statistics from theatre notes and comparison with baseline data.
5. Evaluation of the barriers to the use of the SSC.	How will the barriers to the use of the SSC be evaluated?	Barriers to SSC usage identified.	Transcription of notes from individual interviews and focus group discussions (FGDs).	Interviews and FGDs
6. Training of HCPs and QA managers on SSC usage and implementation.	How will HCPs be trained?	Number of training sessions of HCPs at the hospital: Information sharing meeting Discussion of case studies	Register of training sessions	Review of records
7. Missing information	How will missing information on the SSC be minimised?	Percentage of incomplete SSCs. Number of training sessions by QA managers with staff involved in theatre.	SSCs used in monthly audits. Register of sessions by the QA managers.	Review of SSCs as part of the monthly audit. Register of training sessions addressing the gap in records.

Note: The evaluation utilised a mixed-method approach.

QA, quality assurance; HCP, healthcare providers; CDs, caesarean deliveries; SSC, World Health Organization Caesarean Surgical Safety Checklist; M&M, morbidity and mortality.

The answers to the questions are the indicators. Table 1 outlines the data matrix used for the SSC implementation at a hospital.

## Sustaining change

As the change leader, one needs to maintain the momentum and energy to ensure that the change is sustained. Many organisations revert to old operational processes without sustained efforts to retain the change culture. Strategies to maintain the new working culture are to continue dealing with denial and resistance by maintaining communication and dealing with problems efficiently. It is also essential to assist individuals committed to innovative solutions in managing issues. The new system may require redesign if it does not work effectively and efficiently. Be prepared to tweak the new system. Endorse the positive aspects of the new approaches publicly and stress how the new processes have solved old problems. Regular communication with stakeholders is essential. Publish organisational rewards and efficient use of resources through the network.^1^

## Conclusion

Leading change management is a complex activity, so meticulous planning using an inclusive process while being aware of the organisational turmoil and emotional responses involved in implementing change is needed. Logical planning using specific, measurable, achievable, relevant activities with definite time frames will facilitate change management. Build an evaluation framework and devise strategies for ongoing sustainability to ensure long-term success.
